# Effect of Microwave and Ultrasound during the Killing Stage of the Curing Process of Vanilla (*Vanilla planifolia*, Andrews) Pods

**DOI:** 10.3390/foods12030469

**Published:** 2023-01-19

**Authors:** Oscar Antonio-Gutiérrez, Isidro Pacheco-Reyes, Luicita Lagunez-Rivera, Rodolfo Solano, María del Pilar Cañizares-Macías, Gerard Vilarem

**Affiliations:** 1 Laboratorio de Extracción y Análisis de Productos Naturales Vegetales, Centro Interdisciplinario de Investigación para el Desarrollo Integral Regional Unidad Oaxaca, Instituto Politécnico Nacional, Hornos 1003, Oaxaca 71230, Mexico; 2 Departamento de Química Analítica, Facultad de Química, Universidad Nacional Autónoma de Mexico, Ciudad Universitaria, Ciudad de Mexico 04510, Mexico; 3 Laboratoire de Chimie Agro-Industrielle, Université de Toulouse, INP-ENSIACET, 31030 Toulouse, France

**Keywords:** microwave, ultrasound, vanillin, *Vanilla planifolia*

## Abstract

The curing process (CP) of *Vanilla planifolia* pods, which is a long and tedious process, is necessary to obtain the natural vanilla extract. This research evaluated the application of microwave (M) and ultrasound (U) during the “killing” stage of the CP and its effect on vanillin content and β-glucosidase activity. The pods were immersed in a container with water or with moistened samples for the M treatments. In U treatments, the pods were immersed in an ultrasonic bath. After this stage, the samples were subjected to an additional U treatment. The results show that the application of these technologies significantly improves vanillin yield (*p* < 0.05) and the curing time is reduced to 20 days. U treatments subjected to additional sonication at 38 °C obtain more than double the yield of vanillin regarding control. The effect of M and U on cell structure damage increases with additional sonication, but at 15 min, β-glucosidase inactivation decreases the final yield. Disposition of samples in M also affects the final vanillin content. There is no significant correlation between β-glucosidase and vanillin in the different treatments. The application of M and U with the appropriate parameters reduces the CP time without affecting the compounds of interest.

## 1. Introduction

The natural flavor of vanilla encompasses a wide variety of aromatic compounds formed after the curing process (CP) of green vanilla pods and these commercially important aromatic compounds are obtained through the use of extraction systems [1]. Vanillin, which is the most representative compound, is mainly present in the non-volatile glycosylated form of glucovanillin in green vanilla pods [2]. However, the composition of the pods changes after being subjected to the curing process. This process usually has four stages: killing, sweating, drying, and conditioning [3]. The killing step is intended to promote enzymatic reactions, destroying the cell structure and stopping the aging of the pods [4]. Regarding the curing method, it has been shown that the conditions of this first stage have a significant effect on the final vanillin content. Yeh et al. [5] analyzed different conditions of time and temperature during killing in a traditional CP and found that the highest percentage of vanillin (79%) was obtained with treatments at 65 °C for 1 min, and the lowest percentage (62%) was observed with treatments at 80 °C for 10 s each. They also compared two different sweating temperatures and this stage did not exert different effects on the aroma. Nevertheless, this CP has several disadvantages such as low yields and being a laborious process, among others, resulting in limited commercial availability [6]. Therefore, it is of interest to evaluate possible alternatives to improve this process.

On the other hand, in recent years, there has been a worldwide interest in natural, healthy, and minimally processed foods, especially with regards to ingredients such as flavorings [7]. Additionally, the industry must ensure the use of technologies with minimal environmental impact. To increase production efficiency and reduce environmental pollution, alternatives to conventional transformation, preservation, and extraction procedures, such as ultrasound and others, have been studied [8,9].

Microwave (M) and ultrasound (U) are inexpensive and valuable tools that are being investigated in different applications. Besides saving energy, these green techniques offer more opportunities such as the development of new functional products or extracting more powerful functional bioactive compounds [10,11]. To the best of our knowledge, the application of M and U technologies during the CP have not been studied. These technologies have been used primarily for the extraction of vanillin from cured vanilla beans with encouraging results [12,13]. About this, Dong’s research [14] compared ultrasound-assisted extraction, pressure-assisted extraction, and microwave-assisted extraction of vanillin from *Vanilla planifolia*, where microwave-assisted extraction showed the strongest extraction power, shortest time, and highest antioxidant activity. The objective of the present investigation was to evaluate a curing methodology under controlled conditions that possibly reduced the processing time and increased, as much as possible, the vanillin content of cured *Vanilla planifolia* pods by the application of M and U technologies during the first stage of the CP. It is expected that with the results obtained, the curing process will not require long steps or specialized handling.

## 2. Materials and Methods

### 2.1. Vanilla Pods

The vanilla (*Vanilla planifolia* Andrews) pods were harvested at physiological maturity (15–20 cm long and 1–1.5 cm wide, green blossom-end-yellowish) in the community of Nuevo Progreso, San Juan Colorado, Oaxaca, Mexico. The samples were stored at 25 °C in sterile polystyrene bags until further use.

### 2.2. Chemicals and Reagents

The p-nitrophenyl-β-glucopyranoside (pNPG) and vanillin were obtained from Sigma-Aldrich Co., Ltd. (Darmstadt, HE, Germany). Other chemicals and reagents were obtained from J.T. Baker^®^ (Waltham, MA, USA).

### 2.3. Curing Methodology

**Figure 1:**
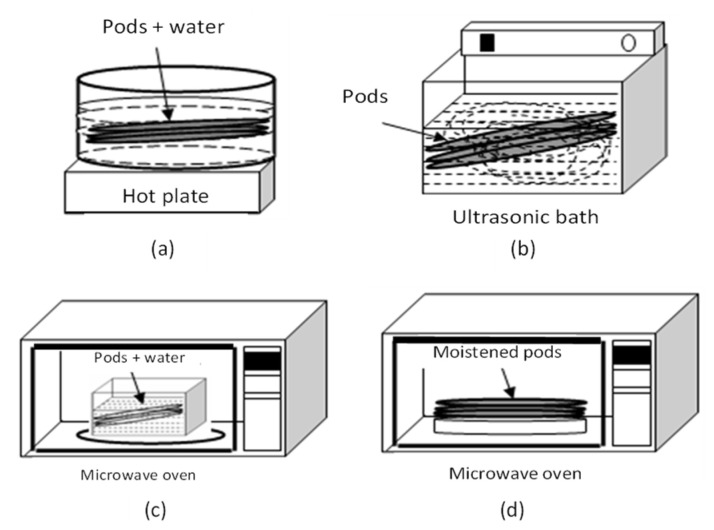
Equipment used during the “killing” stage of the CP: (**a**) water bath treatment (W), (**b**) ultrasound treatment with pods immersed in an ultrasonic bath with water (U1), (**c**) microwave treatment with pods immersed in a container with water (M1), (**d**) microwave treatment with moistened samples (M2).

The use of M and U was analyzed at the stage of “killing”, which is considered the first stage in the CP. The pods were heated to 65 °C with different treatments (water bath (W), M, and U) and combinations of M and U. For the W treatment, the pods were immersed in a container with water and heated for 3 min at 65 °C. For M treatment, first (M1), the pods were immersed in a container with water in a microwave oven (Samsung MW8574W, Samsung Electronics Co., Seoul, Republic of Korea), and second (M2), moistened samples were placed on the microwave oven plate. The microwave oven worked at a fixed power of 850 W. In both cases, the samples were on the rotating microwave plate and irradiated until reaching 65 °C, which was reached at 2.5 min and 30 s, respectively. In the case of U treatment, the samples were immersed in an ultrasonic bath (U1) (Cole-Parmer, Model 8890, Vernon Hills, IL, USA) with water at 65 °C for 3 min. The ultrasonic equipment worked at a fixed power of 100 W and 42 kHz. The disposition of the sample and the equipment used is shown in Figure 1.

Immediately after the “killing” stage, all the samples were subjected to an additional ultrasound treatment (U2) at different times (0, 5, 10, and 15 min) and at 38 °C in the ultrasonic bath. Finally, the sweating, drying, and conditioning stages of the traditional curing process were replaced by drying at 38 °C. The pods, wrapped in cotton cloth and inside high-density polyethylene bags, were placed in a drying oven (Riossa, Model 181932, Mexico City, Mexico) until achieving a 70% loss in their weight. The drying stage was carried out for all treatments and lasted 40 days. The experimental model adopted in the present study is shown in Table 1. In each treatment, a total of 20 pods was used. All treatments were performed in triplicate.

### 2.4. Extraction and Quantification of Vanillin

A total of 3 g of finely cut pods was placed in 50 mL of absolute ethanol in a 100 mL container and allowed to stand for 24 h. Then, 100 µL of the extract was diluted with ethanol, Britton–Robinson solution, and distilled water until reaching a volume of 50 mL. To analyze the amount of vanillin, a calibration curve was elaborated, with vanillin dilutions of 0.001–0.007 g/L, obtained from a standard solution of 1.0 g/L, according to the methodology developed by Valdez-Flores [15]. The absorbance of vanillin was measured in a UV–vis spectrophotometer (Shimadzu UV-1601, Shimadzu Co., Kyoto, Japan). From the standard curve, a directly proportional relationship was obtained between the concentration of diluted extracts and the percentage of vanillin per 100 g of beneficiated pod.

### 2.5. Enzymatic Activity

The extraction and quantitative analysis of the β-glucosidase activity was determined as described by Odoux et al. [16], with a little modification. The pods were finely chopped, and 0.1 M sodium phosphate (pH 7) was added in a weight: volume ratio (1:10). The pulp obtained was filtered through a paper (Whatman No. 42) and 50 µL of the filtrate was immediately diluted in 10 mL of the same sodium phosphate buffer. A total of 500 µL of the obtained dilution and 500 µL of the pNPG substrate were incubated for 20 min at 40 °C. The reaction was stopped with 2.5 mL of 0.5 M sodium hydroxide; immediately, the absorbance at the wavelength of the p-nitrophenol calibration curve was measured. Results were expressed as nkatal per g of fresh pod (1 nkatal of β-glucosidase activity is the amount of enzyme that hydrolyses 1 nmol of substrate per s).

### 2.6. Microscopy

The cell damage generated by the treatments was observed with a light microscope (ZEISS Axiolab 5, Carl Zeiss, Jena, Germany) for brightfield with photographic camera at 10× magnification. Thin cross sections of the most representative pods from the different experiments were placed between a cover and an object slide with distilled water or immersion oil for further analysis.

### 2.7. Statistical Analysis

Data were expressed as mean ± standard error. The experiments were performed in triplicates. Minitab^®^ 17 (Minitab Inc., State College, PA, USA) software was used to conduct an analysis of variance (ANOVA) and Tukey´s test of the data (*p* < 0.05).

## 3. Results and Discussion

### 3.1. Effect of Microwave and Ultrasound on Vanillin Yield

The results of the vanillin yield of the different treatments are shown in Table 2. As can be seen, the yields are obtained in a range from 0.50 to 3.13%. In most of the treatments, the highest yield is obtained 20 days after starting the curing process. The treatments W10, M1-10, M2-5, U1-5, and U1-10 have 3.04, 3.02, 3.11, 3.13, and 3.12% of vanillin yield, respectively, at 20 days of curing.

Traditional curing is a process that can last up to 6 months and the vanillin content in cured pods varies depending on different factors such as the type of curing or even the place of origin of the pods, and is usually presented in a concentration of 1.0–3.0% (dry weight) [17]. It is observed that with the use of M and U technologies in the appropriate conditions this time is significantly reduced (around 90%), compared to traditional curing, obtaining a vanillin yield greater than 3%. Also, in the treatments W10, M1-10, M2-5, U1-5, and U1-10, the yield is more than double that in the case of the control (1.26%). According to ANOVA, the application of these technologies significantly improves the vanilla yield (*p* < 0.05), with the treatments with an additional ultrasound treatment (U2) at 5 and 10 min providing the highest yield.

Sreedhar et al. [18] analyzed the effects of pretreatments on flavor formation in vanilla beans during a traditional CP at 38 °C for 40 days. They obtained 2.13% at 20 days of curing. In our case, the application of the ultrasound treatment during the killing stage generates a higher yield of vanillin. A possible explanation for this higher yield may be that sonication ruptured many of the cell membranes, thus, β-glucosidase and vanillin precursor substrates are brought into greater contact. This cellular decompartmentalization that increases the release of compounds has also been observed in treatments with high hydrostatic pressure in the curing of vanilla pods [19]. During the CP, significant differences (*p* < 0.05) between the different curing days are observed. Only the treatments M1-10, M2-5, and U1-5 maintain vanillin yields of between 1.67 and 2.05%. A number of different reactions, chemical and physical, can lead to the loss of vanillin [20]. However, the drying process represents the most important physical effect, since a prolonged drying time can lead to a decrease in the vanillin content [21]. In addition, during the final stage of curing, some biochemical reactions are generated, such as oxidative degradation, where vanillin can be oxidized, and losses can occur. [22]. Conventionally cured beans contain between 1 and 2% of vanillin through a process that takes several months; it is notable that the use of microwaves and ultrasounds in the killing stage of curing allowed us to obtain a higher yield of vanillin in less time, reaching 3.13% in just 20 days in the U1-5 treatment.

### 3.2. Effect of Microwave and Ultrasound on β-Glucosidase Activity

**Figure 2:**
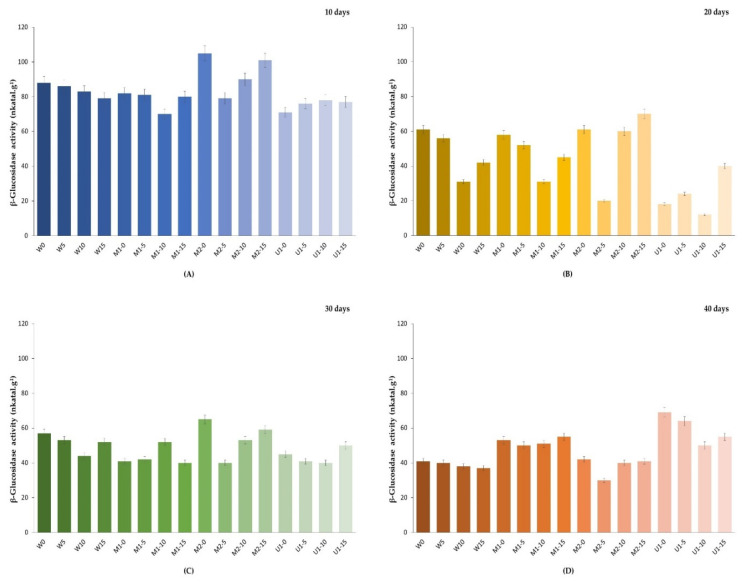
Changes in β-glucosidase activity during the curing process of vanilla pods treated with microwave and ultrasound technologies (nkatal.g^−1^ fresh weight). W, pods immersed in a container with water. M1, pods immersed in a container with water in a microwave oven. M2, moistened pods placed on the microwave oven plate. U1, pods immersed in an ultrasonic bath. All experiments with additional ultrasound treatment at different times (0, 5, 10, and 15 min). (**A**) After 10 days of curing, (**B**) after 20 days of curing, (**C**) after 30 days of curing, and (**D**) after 40 days of curing.

The activity value of β-glucosidase in the ripe pods before the curing process was 1186.11 nkatal.g^−1^. Green vanilla pods are rich in β-glucosidase activity and this enzyme and glucovanillin are sequestered in different regions of plant tissue, therefore, it is necessary to induce cell destruction to release the vanilla aroma that develops during enzymatic hydrolysis of glycosylated aromatic precursors [23]. Although after 10 days of curing, greater enzyme activity is observed in treatments M2-0, M2-10, and M2-15 (Figure 2), none of these treatments present high yields of vanillin, which confirms that a greater cellular decompartmentalization is needed to increase the release of compounds.

It is also observed that the enzyme activities during curing show large differences, since increases and decreases are observed throughout the CP for some treatments. For example, in the ultrasound treatments (U1-0, U1-5, U1-10, and U1-15T15), in the first days a constant decrease is observed, but by day 40 the activity increases almost to the initial values of the curing. This behavior was previously reported by Silva et al. [24]. They confirmed that glucovanillin hydrolysis continued at very advanced stages of the curing.

It is also observed that after the killing stage, β-glucosidase activity decreases by more than 90% for all treatments. Márquez et al. [25] concluded that the “killing” stage of the traditional CP (water at 60 °C, pH 6.0, for 3 min) decreased the enzymatic activity to 50%, which means that half of the β-glucosidase came into contact with the glucovanillin.

In our study, there is no significant correlation (*p* > 0.05) between β-glucosidase and vanillin yield in the different treatments, and although enzymatic activity is present after 20 days of curing, vanillin yield decreases by up to 50% for most treatments at the end of the curing. A possible explanation for this behavior is that aromatic compounds may be lost not only by sublimation or co-evaporation, but also by chemical degradation (oxygen concentration function or temperature) [26]. In the case of ultrasound treatments, sonication causes oxidation and the formation of free radicals, for example, simple sonication of pure water results in the formation of hydroxyl radicals [27]. It is estimated that by irradiating more ultrasound energy, greater intracellular damage is caused, but also other undesirable biochemical reactions are promoted that decrease the yield of vanillin. When the intensity of ultrasound treatment is greater than 10 min, the yield of vanillin decreases, although there are variations in the enzymatic activity.

### 3.3. Micrographs of the Cellular Structure of Pods Treated with Microwave and Ultrasound

**Figure 3:**
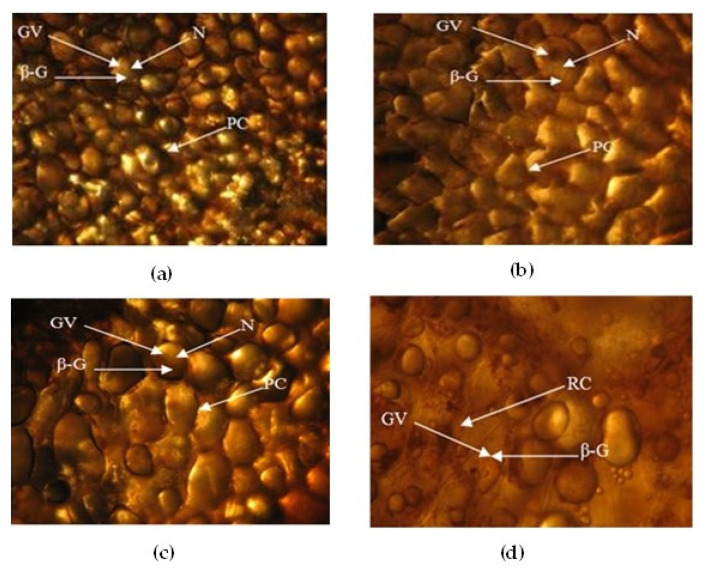
Effect of microwave and ultrasound on the cellular structure of vanilla pods during the killing stage of curing process: (**a**) control (W0); (**b**) M1-0: microwave treatment with pods immersed in a container with water without additional ultrasound treatment; (**c**) M2-0: microwave treatment with moistened samples without additional ultrasound treatment; (**d**) U1-15: ultrasound treatment with pods immersed in an ultrasonic bath with water with an additional ultrasound treatment of 15 min. N: nucleus; PC: cell wall; GV: glucovanillin; β-G: β-glucosidase.

To compare the damage caused to cell structures between a traditional curing and one with M and U, micrographs were taken of the control (W0), M1-0, M2-0, and U1-15. The cellular tissue and the damage generated by these treatments are shown in Figure 3.

In the control treatment, the damage is minimal since the cells wall retain well-defined parenchyma (Figure 3a). However, this stage contributes to the cessation of the vegetative life of the vanilla pods and allows contact between enzymes and substrates [28]. It is also observed that the cellular damage of treatments M1-0, M2-0, and U1-15 is significant (Figure 3b–d, respectively), with the cells wall presenting an important collapse in the U1-15 treatment, with evident damage to the cellular content. Sonication ruptures many of the cell’s membranes and cell walls weaken and fracture. The effect of microwaves and ultrasound on cellular structure increases with longer treatment time and, unlike what was found by Mariezcurrena et al. [29], where the structural change in the pod subjected to hot water treatments shows that neither the outer nor the inner regions of the fruit wall suffer important structural changes during the first three days, in our treatments, the damage is immediate. However, in the U1-15 treatment, the vanillin yield is not optimal. It is worth mentioning that in the case of M2 treatments, some pods exploded, releasing the internal content; this was due to uneven microwave heating, which can cause localized overheating [30]. Despite this, the vanillin content is higher than the control.

The loss presented in the U1-15 treatment is also due to the fact that the application of sonication at that intensity inactivates some enzymes, because their shock waves cause cavitation and the acoustic flow alters the secondary and tertiary structure of the enzymes, causing the loss in their biological activity [31]. The most appropriate procedure for the killing stage with green technologies is achieved by producing a rapid disassembly of the cell membrane without inhibition of the native enzyme, which is with treatments of no more than 10 min of sonication. In the “killing” stage, the disruption of cell structure is an important part of the curing process, since enzymatic reactions are initiated at this stage [32]. Therefore, to obtain the optimal vanillin yield in this investigation, the U2 treatment should not exceed 10 min and the same effect can be observed in microwave treatments. Future research is recommended that involves different variables such as power and time, among others, and both M and U to ensure optimal performance of the vanillin content. Currently, most of the vanillin is obtained by chemical synthesis of the lignin but petro-based resources involve some problems such as contamination [33]. In this investigation, the use of microwave and ultrasound technologies made it possible to reduce curing process time and obtain adequate vanillin yields, with the advantage that these technologies are respectful of the environment.

## 4. Conclusions

The use of microwaves and ultrasounds during the initial stage of CP generates a yield of vanillin comparable to, and even greater than, that obtained by a traditional curing method. The process time is reduced to 20 days compared to a traditional CP, which can take more than 5 months. Also, after the killing stage, no tedious handling and no skilled labor are required with the proposed curing methodology. Therefore, this curing methodology has several advantages over traditional treatment. The damage to the cellular tissue is greater in the ultrasound treatments. Although in the killing stage it was possible to use different techniques, an additional stage where sonication is supplied was necessary to obtain the adequate yields at certain optimal time (5 and 10 min). Additionally, the irradiation times are important, so future studies will be required to analyze different parameters of the process, such as amplitude and power, in order to optimize and improve curing. Due to the encouraging results obtained, it is also necessary to evaluate the effect of these technologies on other important compounds or enzymes of interest.

## Figures and Tables

**Table 1:** Experiments analyzed during the CP.

Treatment	“Killing” at 65 °C				U2 ^5^ at 38 °C			
	W ^1^	M1 ^2^	M2 ^3^	U1 ^4^	0 min	5 min	10 min	15 min
W0	✓				✓			
W5	✓					✓		
W10	✓						✓	
W15	✓							✓
M1-0		✓			✓			
M1-5		✓				✓		
M1-10		✓					✓	
M1-15		✓						✓
M2-0			✓		✓			
M2-5			✓			✓		
M2-10			✓				✓	
M2-15			✓					✓
U1-0				✓	✓			
U1-5				✓		✓		
U1-10				✓			✓	
U1-15				✓				✓

^1^ W, water bath treatment; ^2^ M1, microwave treatment with pods immersed in a container with water; ^3^ M2, microwave treatment with moistened samples; ^4^ U1, ultrasound treatment with pods immersed in an ultrasonic bath with water; and ^5^ U2, additional ultrasound treatment at different times (0, 5, 10, and 15 min).

**Table 2:** Vanillin concentrations (% of dry weight of the pod) from cured pods treated with M and U technologies.

Treatment	10 Days	20 Days	30 Days	40 Days
Control (W0)	0.50 ± 0.02 ^a,A^	1.26 ± 0.12 ^a,B^	1.15 ± 0.09 ^a,C^	1.16 ± 0.13 ^a,C^
W5	0.70 ± 0.05 ^b,A^	1.38 ± 0.22 ^b,B^	1.56 ± 0.12 ^b,C^	1.30 ± 0.36 ^b,D^
W10	1.25 ± 0.10 ^c,A^	3.04 ± 0.08 ^c,B^	2.17 ± 0.21 ^c,C^	1.53 ± 0.08 ^c,D^
W15	1.11 ± 0.06 ^d,A^	2.28 ± 0.15 ^d,B^	2.01 ± 0.35 ^d,C^	1.36 ± 0.09 ^b,D^
M1-0	1.01 ± 0.02 ^e,A^	1.39 ± 0.14 ^b,B^	1.40 ± 0.19 ^e,B^	1.44 ± 0.67 ^d,C^
M1-5	1.77 ± 0.01 ^f,A^	1.55 ± 0.07 ^e,B^	1.48 ± 0.32 ^e,B^	1.55 ± 0.45 ^c,C^
M1-10	2.11 ± 0.02 ^g,A^	3.02 ± 0.19 ^c,B^	2.21 ± 0.90 ^f,C^	1.70 ± 0.06 ^e,D^
M1-15	1.79 ± 0.03 ^f,A^	1.86 ± 0.21 ^f,B^	1.45 ± 0.28 ^g,C^	1.66 ± 0.01 ^e,D^
M2-0	1.13 ± 0.06 ^d,A^	1.66 ± 0.01 ^g,B^	1.78 ± 0.22 ^h,C^	1.19 ± 0.40 ^a,D^
M2-5	1.57 ± 0.07 ^h,A^	3.11 ± 0.05 ^h,B^	2.50 ± 0.40 ^i,C^	2.05 ± 0.11 ^f,D^
M2-10	1.18 ± 0.08 ^i,A^	2.27 ± 0.14 ^d,B^	2.52 ± 0.05 ^i,C^	1.72 ± 0.16 ^e,D^
M2-15	0.87 ± 0.12 ^j,A^	2.09 ± 0.21 ^i,B^	1.81 ± 0.01 ^h,C^	1.22 ± 0.21 ^b,D^
U1-0	1.27 ± 0.10 ^c,A^	1.85 ± 0.02 ^f,B^	1.46 ± 0.03 ^g,C^	1.28 ± 0.14 ^g,D^
U1-5	1.44 ± 0.11 ^k,A^	3.13 ± 0.08 ^h,B^	2.68 ± 0.13 ^j,C^	1.67 ± 0.21 ^e,D^
U1-10	1.39 ± 0.09 ^l,A^	3.12 ± 0.17 ^h,B^	2.35 ± 0.16 ^f,C^	1.50 ± 0.04 ^c,D^
U1-15	1.31 ± 0.08 ^m,A^	1.77 ± 0.06 ^i,B^	2.11 ± 0.30 ^c,C^	1.38 ± 0.05 ^b,D^

All data are expressed as mean ± standard deviation, n = 3. Data followed by different lowercase letters within each column are significantly different according to Tukey test at *p* < 0.05. Data followed by different capital letters in same row are significantly different according to Tukey test at *p* < 0.05. W, pods immersed in a container with water. M1, pods immersed in a container with water in a microwave oven. M2, moistened pods placed on the microwave oven plate. U1, pods immersed in an ultrasonic bath. All experiments with additional ultrasound treatment at different times (0, 5, 10, and 15 min).

## Data Availability

All generated data are included in this article.
